# Responses of the bacterial community of tobacco phyllosphere to summer climate and wildfire disease

**DOI:** 10.3389/fpls.2022.1050967

**Published:** 2022-12-21

**Authors:** Zhenhua Wang, Changwu Fu, Jinyan Tian, Wei Wang, Deyuan Peng, Xi Dai, Hui Tian, Xiangping Zhou, Liangzhi Li, Huaqun Yin

**Affiliations:** ^1^ Zhangjiajie Tobacco Company of Hunan Province, Zhangjiajie, China; ^2^ Yongzhou Tobacco Company of Hunan Province, Yongzhou, China; ^3^ School of Minerals Processing and Bioengineering, Central South University, Changsha, China; ^4^ Key Laboratory of Biometallurgy of Ministry of Education, Central South University, Changsha, China

**Keywords:** tobacco, phyllosphere, bacterial community, high-throughput sequencing, molecular ecological networks, neutral community model

## Abstract

Both biotic and abiotic factors continually affect the phyllospheric ecology of plants. A better understanding of the drivers of phyllospheric community structure and multitrophic interactions is vital for developing plant protection strategies. In this study, 16S rRNA high-throughput sequencing was applied to study how summer climatic factors and bacterial wildfire disease have affected the composition and assembly of the bacterial community of tobacco (*Nicotiana tabacum* L.) phyllosphere. Our results indicated that three time series groups (T1, T2 and T3) formed significantly distinct clusters. The neutral community model (NCM) and beta nearest taxon index (betaNTI) demonstrated that the overall bacterial community assembly was predominantly driven by stochastic processes. Variance partitioning analysis (VPA) further showed that the complete set of the morbidity and climatic variables together could explain 35.7% of the variation of bacterial communities. The node numbers of the molecular ecological networks (MENs) showed an overall uptrend from T1 to T3. Besides, *Pseudomonas* is the keystone taxa in the MENs from T1 to T3. PICRUSt2 predictions revealed significantly more abundant genes of osmoprotectant biosynthesis/transport in T2, and more genes for pathogenicity and metabolizing organic substrate in T3. Together, this study provides insights into spatiotemporal patterns, processes and response mechanisms underlying the phyllospheric bacterial community.

## Introduction

The leaf surface on which microorganisms inhabit is called phyllosphere, which is an independent microhabitat of plant foliar surface. Bacteria, fungi, algae and other microorganisms living in these regions are called phyllospheric microorganisms, the structure of which is affected by a range of abiotic and biotic factors, including space (Rastogi et al., 2012) , growth season (Copeland et al., 2015) , climate variation (Medina-Martínez et al., 2015) , solar radiation (Truchado et al., 2017) and disease severities (Zheng et al., 2022) . The phyllospheric area has long been considered an unfavorable habitat for microbial colonization due to long time exposure to solar/ultraviolet radiation, severe diurnal temperature, desiccation, fluctuation of humidity, scouring rainfall, and the scarcity of available nutrients. Recent high-throughput sequencing technologies have enabled the characterization exhaustively the spatio-temporal structure of phyllosphere microbiome. It has also been found that there are a large number of microorganisms inhabiting the phyllosphere, with a density as high as 10^6^-10^7^ cells per square centimeter with multiple biological functions, such as improving plant disease resistance, biocontrol of phytopathogens, enhancing nitrogen fixation, decomposing toxic and harmful substances, and producing plant hormones, volatile organic compounds (VOCs) to promote plant growth (Vorholt, 2012; Xu et al., 2022) . Besides, the phyllosphere also stands for a suitable model system for testing basic principles in ecology since it is amenable for experiments and visual inspection. Such research has implications for fields like plant health and environmental chemistry (Redford and Fierer, 2009; Remus-Emsermann and Schlechter, 2018) .

A thorough understanding of the ecological drivers of phyllospheric community assembly and multitrophic interactions is vital to develop strategies for plant protection. However, many research aspects of phyllospheric microbiome still lag behind in comparison with rhizospheric studies.

Tobacco (*Nicotiana tabacum* L.), is a model plant and important economic crop, and an ideal research object to study plant-microbe interactions under multiple stresses (Xiang et al., 2022). Tobacco is usually grown in summer and harvested at the end of August. Tobacco’s leaves are continually subjected to excessive strong sunlight on summer days, which makes it an ideal material to study the mechanism of bacterial community assembly and succession in the face of strong abiotic stresses (i.e., UV radiation, desiccation and heat), accompanied with pathogen invasion (Bringel and Couée, 2015; Xing et al., 2022). Dai et al. (2022) investigated the spatiotemporal variation of the community tobacco leaves affected by brown spot disease and found that the relative abundance of *Pseudomonas, Sphingomonas*, and *Methylobacterium* increased as tobacco leaves aging gradually. Besides, Liu et al. (2022) found that the inoculation of *Bacillus velezensis* SYL-3 could increase the abundance of beneficial bacteria, (*Pseudomonas* and *Sphingomonas*), while suppress the pathogens *Alternaria alternata* and tobacco mosaic virus (TMV).

In the current study, we have tried to (i) elucidate successions of the taxonomic and functional profile in the phyllospheric bacterial community under abiotic and biotic stresses of three time periods T1, T2 and T3 in summer (corresponding to the June, July and August of the year 2021), (ii) investigate which members in different bacterial community conferred positive effects on the plant upon abiotic and biotic stresses, and (iii) compare the networks of different period to provide insights into the key taxa of communities.

## Results and discussion

### Bacterial community composition and diversity in tobacco phyllosphere

**Figure 1:**
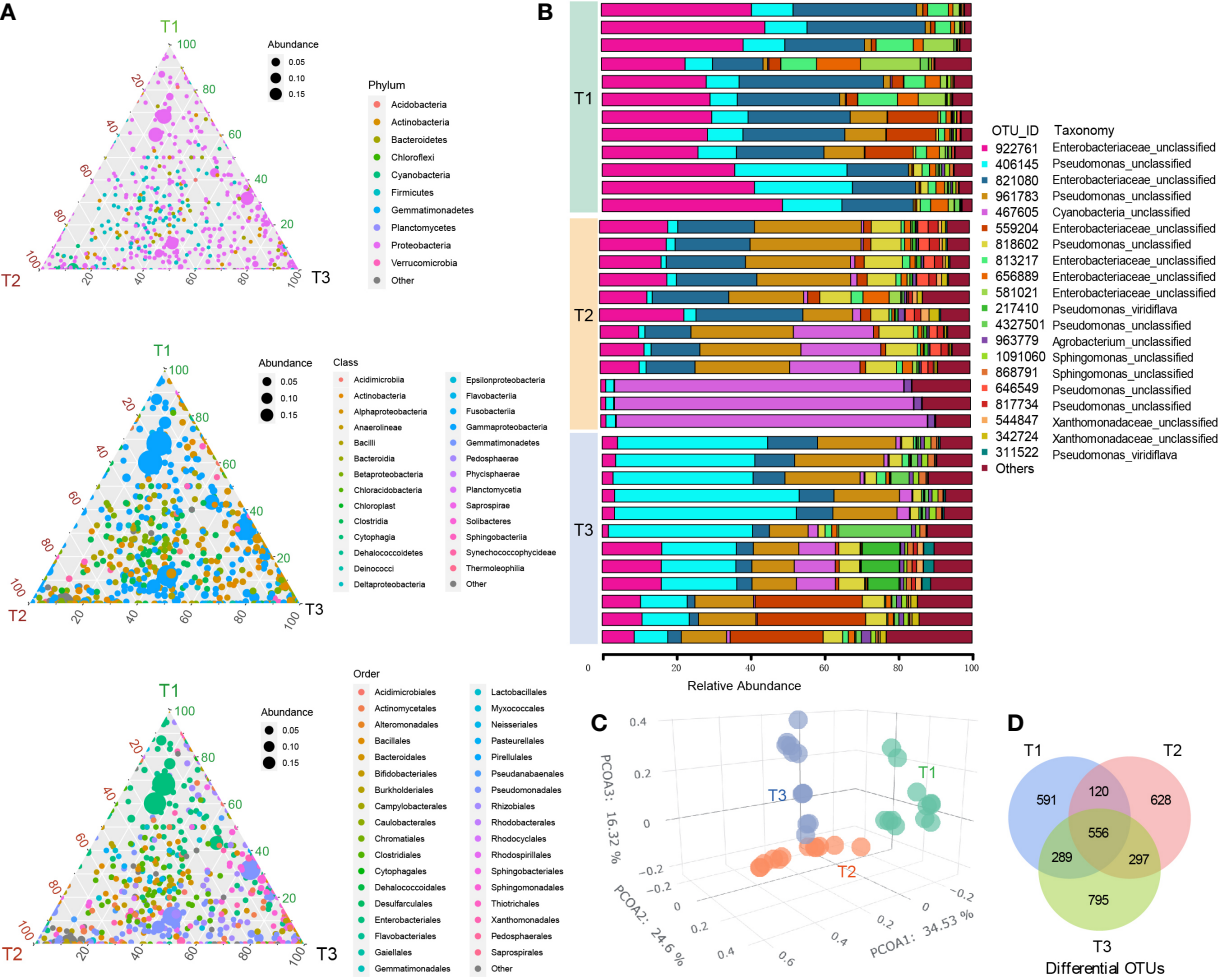
Bacterial community composition and diversity in tobacco phyllosphere. **(A)** The ternary phase diagrams showing the relative abundance and relationships of the different taxonomic categories (top, phylum; middle, class; bottom, order) in the three time series groups (three vertices, T1, T2 and T3). The size of each circle is proportional to relative abundance; the closer the circle is to the vertex, the higher the relative abundance of the group in that group. **(B)** Stack bar chart showing the twenty most abundant OUT taxa in each sample. **(C)** Principal coordinate analysis (PCoA) of Bray–Curtis dissimilarity matrices showing effects of time series (T1, T2 and T3 groups) on the tobacco phyllospheric bacterial community structure. **(D)** Venn diagram depicting number of shared or unique OTUs in each time series group (T1, T2 and T3).

A total of 2,302,148 high-quality paired 16S rRNA sequences and 3,276 operational taxonomic units (OTU) were obtained from 36 tobacco phyllospheric bacterial DNA samples, (average: 63,949; range: 47,651–79,418 reads per sample). The ternary phase diagrams (**Figure 1A**) show the relative abundance and relationships of the different taxonomic categories in the three time series groups (T1, T2 and T3), with the circle’s size and vicinity to the vertex proportional to relative abundance in the respective group. We found that the phylum *Proteobacteria*, class *Gammaproteobacteria* and orders *Pseudomonadales* and *Enterobacteriales* made up the majority of phyllospheric bacterial taxa (**Figures 1A, B**). In comparison, the phyllosphere of other species from the solanaceous family such as tomato (*Lycopersicon esculentum*) is also additionally dominant by *Rhizobium*, *Methylobacterium*, and *Xanthomonas* (Ottesen et al., 2013; Toju et al., 2019).

**Figure 2:**
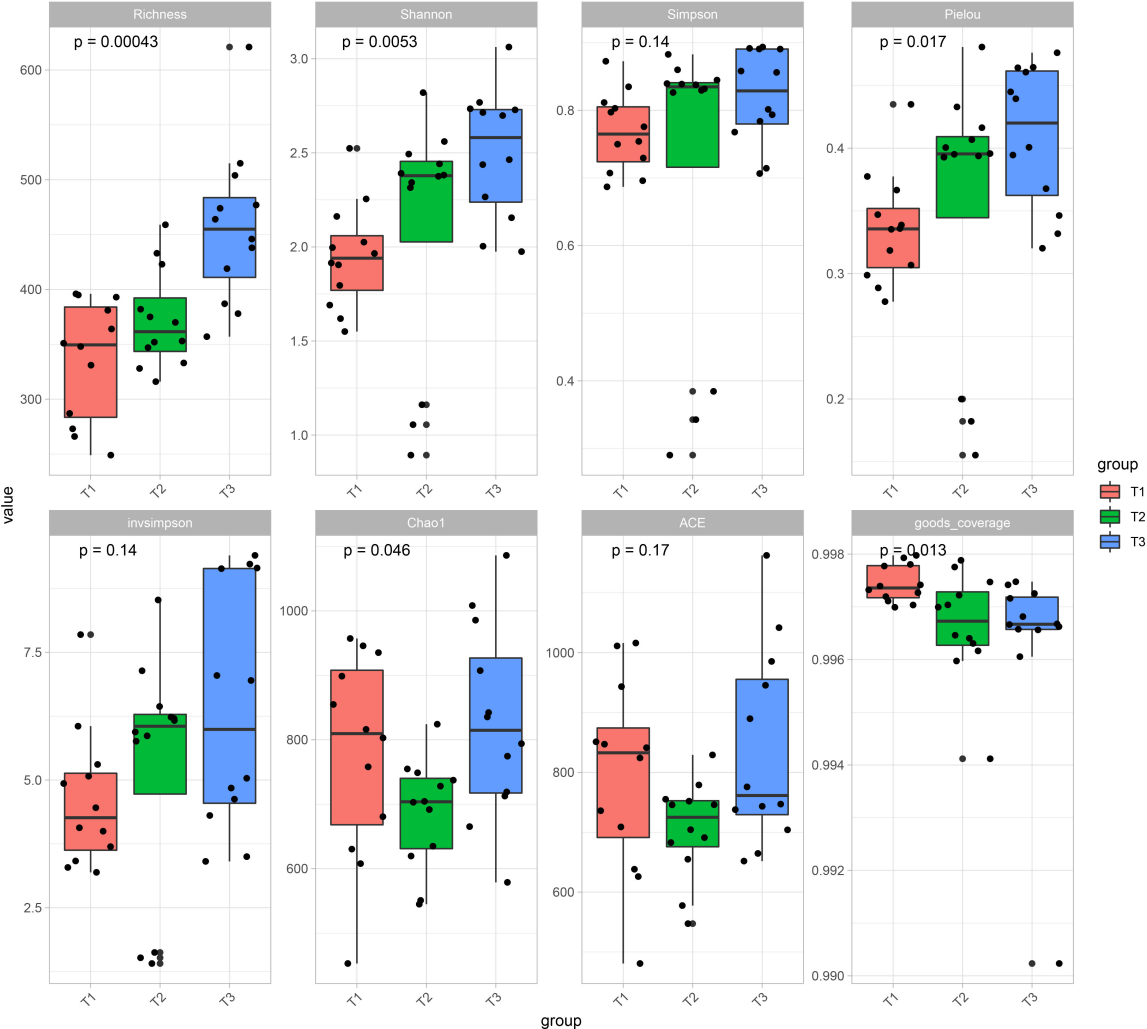
Alpha diversity indexes including Richness, Shannon, Simpson, Pielou, invsimpson, Chao1, ACE, and goods coverage of tobacco phyllospheric bacterial communities in each time series group (T1, T2 and T3).

Principal coordinate analysis (PCoA) of Bray–Curtis distance (**Figure 1C**) revealed that the microbiome from three time series groups (T1, T2 and T3) formed three significantly distinct clusters, indicating that phyllospheric microbiome from different time periods exhibited distinct community compositions. The first three axes together explained 75.4% of the cumulative variation (ANOISM analysis, p <0.001, R=0.597). A total of 556 OTUs are shared across three groups (**Figure 1D**), and T3 group comparatively has more unique OTUs (795), followed by T2 group (628) and T1 group (591). Alpha diversity indexes, including Richness, Shannon, Simpson, Pielou, and invsimpson showed an obvious uptrend from the T1 group to T3 group (**Figure 2**).

**Figure 3:**
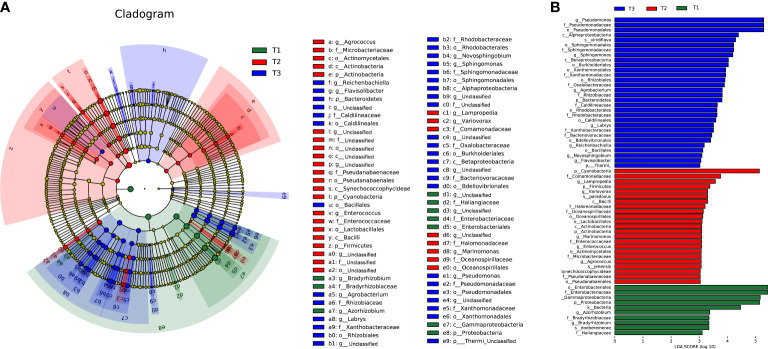
The linear discriminant analysis effect size (LEfSe) analysis at species level of bacterial communities (with LDA score >3.1 and p < 0.05) among T1, T2 and T3 groups presented by **(A)** cladogram **(B)** and distribution histogram.

To determine the changes in bacterial community composition across time series, LEfSe analysis was applied to find the differential taxa in each group. The linear discriminant analysis (LDA) score is positively correlated with the significance of bacterial biomarkers in each group (**Figure 3**). Comparatively, T1 group shows enrichment of bacterial families like *Enterobacteriaceae* (LDA =5.89) and *Haliangiaceae* (LDA =3.12), genra *Azorhizobium* (LDA =3.37) and *Bradyrhizobium* (LDA =3.34), and species *Azorhizobium doebereinerae* (LDA =3.34). Members of *Enterobacteriaceae* in plant phyllosphere are sensitive to abiotic or biotic stresses (Erlacher et al., 2015), whose population sizes decline upon desiccation stress on leaves (Brandl and Mandrell, 2002; Brandl, 2006; Whipps et al., 2008), and they contribute to the overall phyllospheric resistome (Cernava et al., 2019).

At the same time, T2 group shows enrichment of bacterial phylum *Cyanobacteria* (LDA =5.13), *Firmicutes* (LDA =3.37) and *Actinobacteria* (LDA =3.09), class like *Alphaproteobacteria* (LDA =3.59), orders like *Burkholderiales* (LDA =3.57) and *Oceanospirillales* (LDA =3.23). In previous studies, *Firmicutes* (represented by *Bacillus* sp.) and *Cyanobacteria* were considered beneficial to plant growth and pathogen control (Priya et al., 2015; Han et al., 2016; Liu et al., 2022), indicating that plant host might have induced the enrichment of these pathogen antagonists in the phyllosphere during T2 to rescue itself from disease invasion.

Whereas the T3 group shows strong enrichment of genra including *Pseudomonas* (LDA =5.29), *Sphingomonas* (LDA =4.19), *Agrobacterium* (LDA =3.82), and family like *Xanthomonadaceae* (LDA =3.94). Most *Pseudomonas* species are beneficial for plants, producing phytohormones and siderophores to the inhibit pathogens (McSpadden et al., 2001; Hsu and Micallef, 2017) and induce host systemic resistance to improve morphological and biochemical traits of plants at the same time (Kumar et al., 2021). Besides, the abundance of *Pseudomonas* was negatively correlated with the disease index caused by pathogens *Alternaria alternate* (leaf spot or blight disease) tobacco mosaic virus (TMV) both in tobacco (Liu et al., 2022) and tomato (Gupta et al., 2021). Likewise, *Sphingomonas* sp., is a kind of gram-negative, aerobic foliar and phytohormone-producing bacterium capable of protecting plants from foliar diseases caused by *Pseudomonas syringae* (wildfire disease pathogen) *via* substrate competition (Vogel et al., 2012) and various pathogenic fungi (e.g., *Alternaria* and *Arthrinium*) (Enya et al., 2007; Vogel et al., 2012; Luo et al., 2019) and improving plant growth during stress conditions (Asaf et al., 2020). The enrichment of *Pseudomonas* and *Sphingomonas* in T3 might be a”cry for help” strategy of tobacco for the recruitment of microbes upon biotic (pathogen invasion) and abiotic stresses (drought and heat) (Wang and Song, 2022).

**Figure 4:**
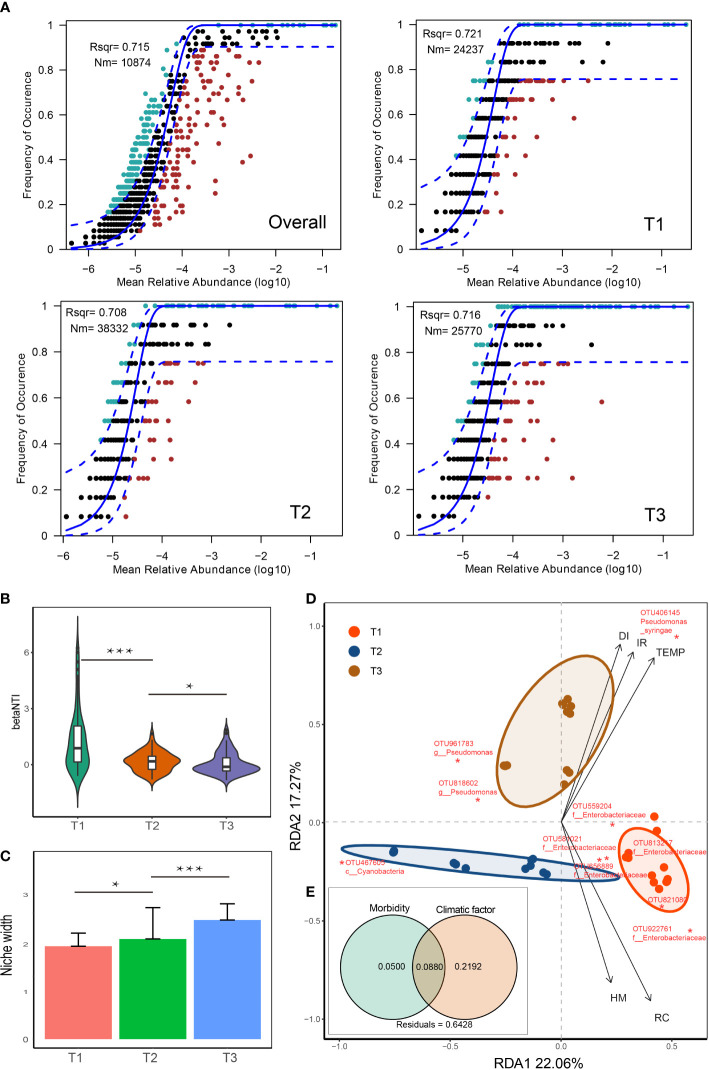
Analyses on factors that impacted the relative abundance and occurrence frequency of microbes in tobacco phyllosphere. **(A)** Fit of the neutral community model (NCM) of community assembly. The OTUs more frequently present than predicted are in cyan, whereas those less frequently are in red. The blue dashed lines represent 95% confidence intervals around the model prediction and the OTUs fallen into the confidence intervals are regarded as neutrally distributed. Nm indicates the meta-community size times immigration, Rsqr indicates the fit to the neutral model. Neutral processes are the part within 95% confidence interval (red) while non-neutral are the parts including above and below prediction (dark green); **(B)** beta nearest taxon index (betaNTI) comparison; **(C)** niche breadth comparison; **(D)** redundancy analysis (RDA) of the relationships between bacterial community in tobacco leaves and environmental variables, including morbidity variables (disease incidence rate: IR, and disease index: DI) and climatic factors (temperature: TEMP, humidity: HM, and rainfall capacity: RC), **(E)** variance partitioning analysis (VPA) showing contributions of morbidity and climatic variables to tobacco phyllospheric bacterial community variation. Asterisks indicate significance; *, p < 0.05; ***, P< 0.001.

The neutral community model (NCM) is a validated method for deducing stochastic processes related to community assembly, which has been helpful in explaining various ecological phenomena (Roguet et al., 2015) . This model could quantify the significance of processes that are not easy to observe directly but might have a great impact on microbial communities (i.e., dispersal and ecological drift). In our study, the neutral community model (NCM) has successfully predicted a large fraction of the relation between the occurrence frequency of OTUs and the relative abundance (**Figure 4A**), with 72.1%, 70.8%, 71.6% and 71.5% of explained community variance for T1, T2, T3 groups and overall, respectively, indicating similar responses of species in different groups to stochastic processes. Besides, the Nm value is relatively higher for T2 group (Nm = 38,332) than T1 group (Nm = 24,237) and T3 group (Nm = 25,770), indicating that the species dispersal was higher in T2 group. Consistently, the majority of betaNTI values of these tobacco phyllospheric bacterial communities fall within -2 and +2 (stochastic process) in all groups (**Figure 4B**). The distributions of betaNTI shifted with a downtrend from higher betaNTI in T1 showing more deterministic community assembly processes (betaNTI > 2) to lower betaNTIs in T2 and T3 predominant with stochastic community assembly processes (-2 < betaNTI < +2). Besides, the estimated niche width of T3 group is significantly greater than that of T2 and T1 (**Figure 4C**). These results suggested that stochasticity was more important than determinism in influencing the tobacco phyllospheric bacterial community

It has been demonstrated that environmental factors (e.g., water, temperature, nutrient and metal concentrations) affect the microbial community composition, which further impact the relative abundance and occurrence frequency of microbes in the neutral or non-neutral distribution . (Liu et al., 2013; Logares et al., 2013; Zhang et al., 2018) Rainfall always concentrates in the summer season in the subtropical area of China. Zhangjiajie region (the sample collection site) generally has the annual precipitation peak in July (corresponding to T2), according to data from national meteorological center of China (http://www.nmc.cn/). Such rainfall abounding conditions, along with accompanying high aerial humidity, could increase the air retention time of microbe (pathogen) and facilitate their passive dispersal across space with higher immigration rate and spores germination on the leaf surface. This might explain why the T2 group has the highest Nm-value in the neutral community model, similar to what have observed in previous studies (Chen et al., 2019) .

### Relationship between bacterial populations and environmental factors

Redundancy analysis (RDA) was further applied to reveal the relationship between phyllospheric bacterial populations and factors (**Figure 4D**). RDA results showed that morbidity variables, including wildfire disease incidence rate (IR) and disease index (DI), are positively correlated with temperature (TEMP) and OTU406145 (*Pseudomonas syringae*, the pathogen of bacterial wildfire disease), whereas they negatively correlated with OTU467605 (*Cyanobacteria*), indicating that *Cyanobacteria* is the potential disease biocontrol agent. Consistently, a previous study also indicated that *Cyanobacteria* was a major phylum on the leaf of tobacco (Xing et al., 2021) and *Cyanobacteria* may play a major role in nutrient cycling and water storage in the phyllosphere (Fürnkranz et al., 2008). Besides, humidity (HM) and rainfall capacity (RC) are consistently and positively correlated with *Enterobacteriaceae* (OTU559204, OTU581021, OTU813217, OTU656889, OTU922761). This is consistent with previous reports that members of *Enterobacteriaceae* in the phyllosphere are capable of rapid reproduction and formation of aggregates under high moisture conditions and are sensitive to fluctuations in water availability on plant surfaces (Brandl and Mandrell, 2002; Brandl, 2006; Whipps et al., 2008).

Overall, the morbidity (IR, DI) and climatic factors (TEMP, HM, RC) have significantly affected the phyllospheric bacterial community. Variance partitioning analysis (VPA) further showed that the complete set of the morbidity and climatic variables together could explain 35.7% of the variation of tobacco phyllospheric bacterial communities, with climatic variables contributing most (**Figure 4E**). Still, the high proportion of unexplained variation in VPA also suggested the potential importance of neutral or stochastic processes during community assembly.

### Molecular ecological networks of phyllospheric bacterial community

**Figure 5:**
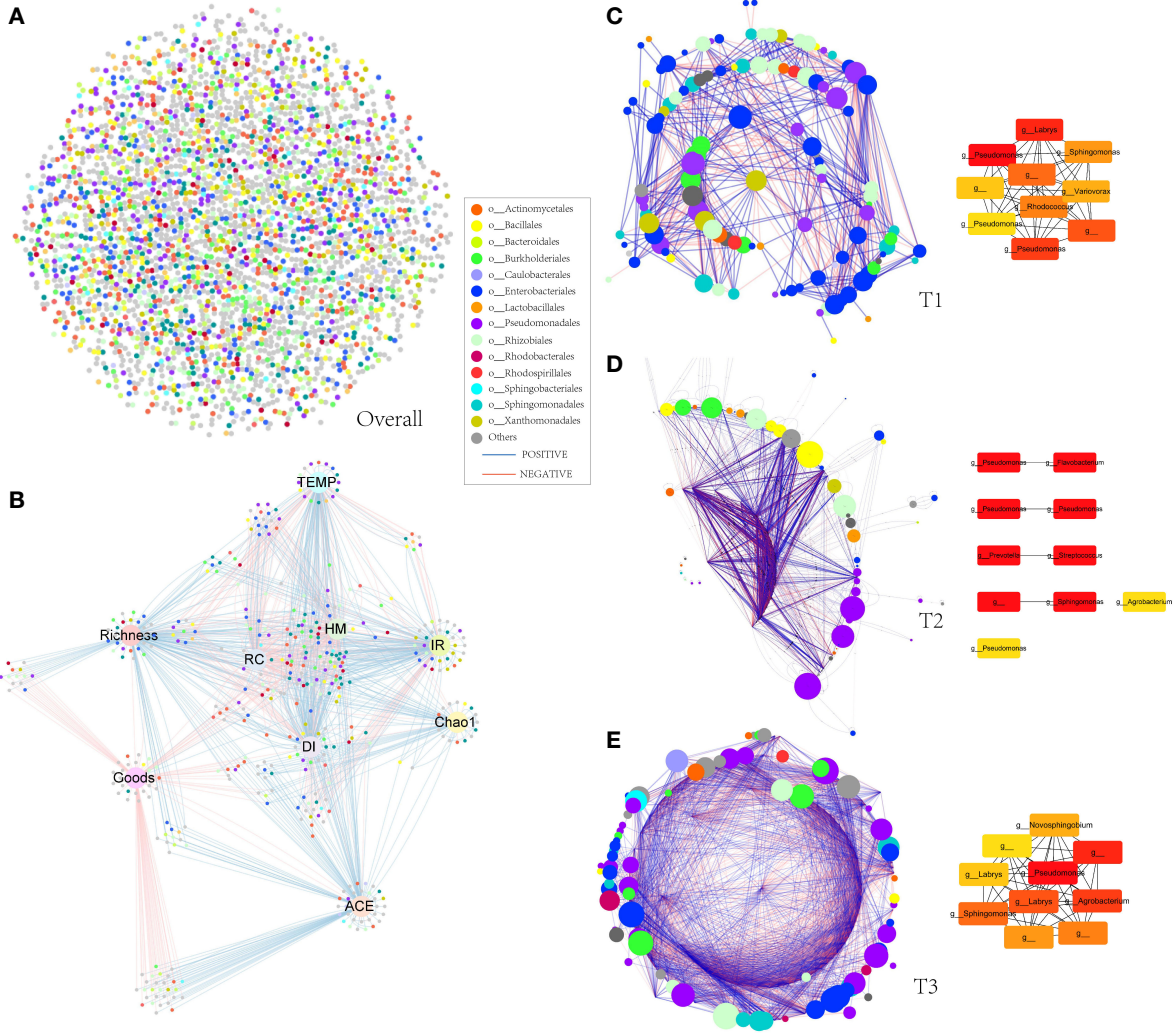
Molecular ecological networks of phyllospheric bacterial community. Each node represents an OTU. The size of each node is proportional to the number of connections (that is, degree) and the colors of nodes represent different order. The links between the nodes indicate strong and significant (*P *< 0.01) correlations. A red line indicates a negative interaction between two individual nodes, while a blue line indicates a positive interaction. **(A)** Overall molecular ecological network; **(B)** spearman correlation between environmental factor, alpha diversity index, and OTUs. **(C)** molecular ecological network of T1 group (left) and predicted keystone nodes (right); **(D)** molecular ecological network of T2 group (left) and predicted keystone nodes (right); **(E)** molecular ecological network of T3 group (left) and predicted keystone nodes (right).

Molecular ecological networks (MENs) were constructed to unravel how the combinations of bacterial wildfire disease and climatic factors have affected microbial interactions across the three time periods (**Figure 5**). The topological properties of the three sub networks were shown in **Table 1**. The node numbers of the phyllosphere networks showed an overall uptrend from T1 to T3. Whereas link numbers increase sharply from T1 (639) to T2 (14,401), followed by a rapid decrease to T3 (3,533). Similar trend is also found in MEN properties like the average number of neighbors, network density (comparison between the edges available in a graph and a graph with all possible edges), network centralization (measure of how much the degree of every node is far from the degree of the highest degree node), and connected components (a maximal set of nodes such that each pair of nodes is connected by a path). This indicated that T2 was more complex, with abundant interactions in a highly connected microbial community, which might also be explained by the rainfall peak at T2 that has provided great growth opportunity for microbes to multiply and make connections as mentioned above. A similar phenomenon of relatively higher connections was also observed in the wilt diseased rhizoplane of tobacco (Tao et al., 2022) . The percentage of positive correlation follow largely a downtrend from T1 (57.4%) to T3 (53.6%), indicating the decrease of cooperative relation. Besides, it is predicted by the program cytoHubba (Chin et al., 2014) that *Pseudomonas* is the keystone taxa and is present in the top 10 important nodes in the MENs from T1 to T3 (**Figure 5**). Other frequently present keystone taxa includes *Sphingomonas* and *Labrys.* These taxa are reported to be dominant in plant phyllosphere and might play important roles in inhibiting plant disease (Liu et al., 2021; Liu et al., 2022).

**Table 1:** Molecular ecological network properties of the three groups.

	T1	T2	T3
Number of nodes	117	191	200
Number of edges	639	14401	3533
Positive link	0.574	0.527	0.536
Negative link	0.425	0.472	0.463
Avg- number of neighbors	10.923	45.73	35.33
Network diameter	6	7	5
Network radius	4	7	3
Characteristic path length	2.677	2.154	2.181
Clustering coefficient	0.409	0.691	0.637
Network density	0.094	0.249	0.178
Network heterogeneity	0.659	0.806	0.64
Network centralization	0.141	0.331	0.206
Connected components	1	7	1

### The function profiles of phyllospheric bacterial community

**Figure 6:**
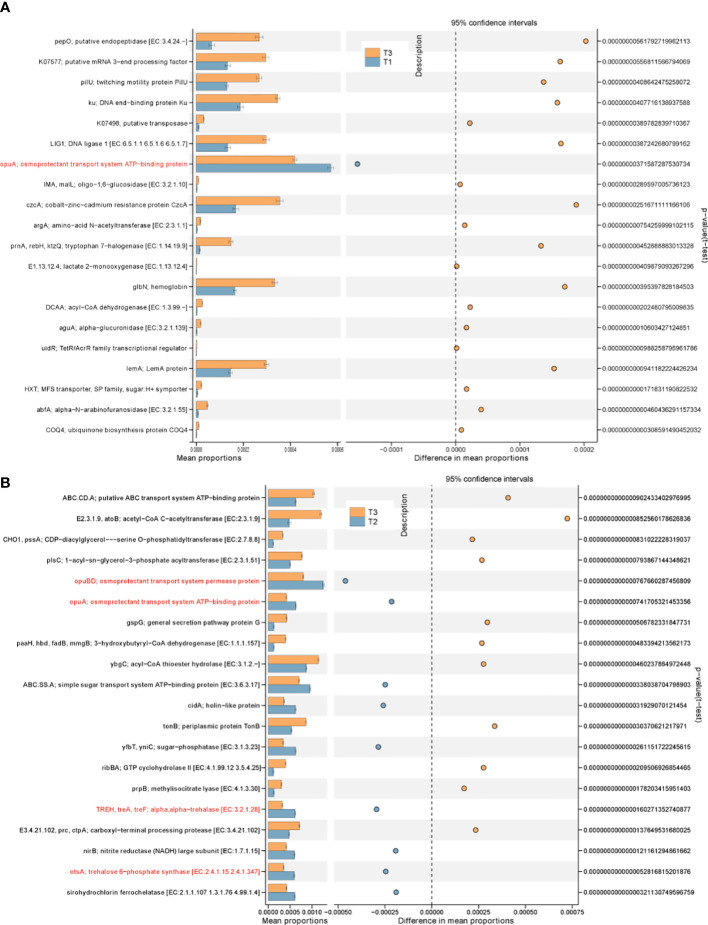
PICRUSt predicted metagenome functions with significant difference in abundance between groups at KO level. **(A)** T1 vs. T3; **(B)** T2 vs. T3.

To assess the putative effect of stress factors on the bacterial community functions of different time periods, metagenome of tobacco phyllospheric bacterial communities were predicted with PICRUSt2 (Douglas et al., 2020) followed by annotation in referring to the KEGG database. A total of 7,557 KOs (KEGG Orthologs) were predicted across three groups, and some of them are significantly different among groups (**Figures 6A, B**). For example, biosynthesis/transport genes of osmoprotectant against osmotic stress (*opuA, opuBD, treA, otsA*) in T2 were predicted to be more abundant than T3 (**Figure 6B**). At the same time, the relative abundance of genes encoding resistance–nodulation–division (RND) type cobalt−zinc−cadmium resistance protein, periplasmic protein TonB, sirohydrochlorin ferrochelatase, twitching motility protein PilU and several metabolic genes for organic substrate (*atoB, paaH, prnA, prpB, ctpA, pepO, aguA, abfA*) is significantly increased in T3. We proposed that these genes enriched in the T3 group were related with the deterioration of wildfire disease, since previous studies have found RND efflux pumps of plant-pathogenic *P. syringae* pathovars essential for *in planta* reproduction and evasion of the host native immune response (Stoitsova et al., 2008) . Besides, TonB-dependent receptor is regarded as a feature shared by phytopathogenic bacteria for the uptake of various carbohydrates under environmental conditions (Blanvillain et al., 2007) . Motility can help wildfire disease pathogen search for favorable sites, facilitate spreading and locate the most preferred site (Haefele and Lindow, 1987) . Toxin such as syringomycin produced by *Pseudomonas syringae* is responsible for pore formation and nutrient leakage through the host cell membrane (Pritchard et al., 2009) , Besides, plants generally produce phenolic substances to counteract increased stress levels (Lucas et al., 2022). This might explain the significant enrichment of organic metabolic genes in the T3 group community.

Collectively, this study has contributed to improving the understanding of the spatiotemporal patterns of tobacco phyllospheric microbiome and shed light on the putative underlying mechanism.

## Materials and methods

### Disease incidence of bacterial wildfire disease

The standards used to examine tobacco bacterial wildfire disease were based on the tobacco pest classification and survey methods (GB/T 23222–2008), P.R. China. Disease incidence was calculated by the percentage of diseased tobacco in each field. Disease index was calculated using the formula:


Disease index (DI) = [∑(r×N)/(n×R)] × 100

r is the disease severity; N is the number of infected tobaccos with a rating of r; n is the total number of tobaccos tested, and R is the value of the highest disease severity in each field. Meteorological data were retrieved from the National Meteorological Center of China (http://www.nmc.cn/).

### Sample collection

The tobacco leaf samples were collected in Gaofeng Town, Cili County, Zhangjiajie City, Hunan Province (29°28′5″N, 110°57′53″E), China, at June (T1), July (T2) and August (T3) of 2021. In the test areas, tobacco fields having typical and serious bacterial wildfire disease levels were selected. In the same plot, with similar conditions, plants with typical symptoms of bacterial wildfire disease were sampled, with three plants per group. The experiment adopted a random block design with three duplicates; the plot area was 90 m^2^. Other field management measures were carried out in accordance with local planting practices. A total of 18 plants were randomly selected from each plot, and the middle leaves of every sixth plant were taken as a sample, which was kept at 4°C and brought back to the laboratory for subsequent foliar microbial DNA extractions.

### DNA extraction and high-throughput sequencing

Fifteen grams of leaf samples obtained from various parts of the leaf surface (avoiding the main and branch veins) using a sterile puncher were added to 50 mL of 0.1% Tween-80 bacterial phosphate buffer (pH 7.0). The samples were then shaken for 30 min at 170 revolutions/min (rpm) and 28°C. The bacterial suspension was then collected, and the leaf samples were washed twice more. The collected suspensions were centrifuged for 15 min (4°C, 10,000 rpm) to pellet the microorganisms. The pellet was suspended in sterile water and washed three times. Finally, the microorganisms were resuspended with 1 mL of sterile water for subsequent DNA extraction. Genomic DNA extraction of foliar microorganisms was performed using the Plant Genomic DNA Kit (Plant Genomic DNA Kit), following the manufacturer’s protocol. We used the primer pair 341F (5’-CCT ACG GGN GGC WGC AG-3’) and 805R (5’-GAC TAC HVG GGTATC TAA TCC-3’) of V3/V4 regions to amplify the 16S rRNA. Amplicons were sequenced by Illumina NovaSeq PE250 platform (LC-Bio Technology Co., Ltd, Hang Zhou, Zhejiang Province, China). The raw sequencing data were deposited in the European Nucleotide Archive database under accession number PRJEB56205.

### Sequencing processing and statistical analyses

Raw sequences were split into sample libraries with perfect matches to barcodes. Low-quality sequences with QC < 20 over a 5-bp window size were trimmed using Btrim (Kong, 2011) , and sequences with a length of < 100 bp were removed. Then, the forward and reverse sequences were spliced together. Any sequences containing ambiguous bases or the incorrect length were removed, and the remaining sequences were compared against the UNITE v8.2 database (Kõljalg et al., 2005) to remove possible chimeras. The length of the sequencing fragment was 200–400 bp. Then, UPARSE (Edgar, 2013) was used to cluster and produce operational taxonomic units (OTUs) at 97% similarity level. To ensure the authenticity of the data, we removed OTUs that were represented by only one sequence in overall data (global singletons). All statistical analyses and calculations were carried out using the R (v 3.6.3) statistical platform (www.r-project.org).

### Network construction

To construct a microbial association network, correlations between pairwise OTUs that were present in more than half of the samples were calculated using the SparCC method (Friedman and Alm, 2012). Only edges with a significant correlation higher than 0.5 (p < 0.01) were retained for network construction. Cytoscape v.3.9.1 (https://cytoscape.org) was used for network visualization. Cytoscape plugin cytoHubba (Chin et al., 2014) with Maximal Clique Centrality (MCC) method was used to predict essential/keystone nodes in the network.

To determine the potential importance of stochastic processes on community assembly, we adopted a neutral community model (NCM) to predict the relationship between OTU detection frequencies and their relative abundance across the wider metacommunity, performed using R (version 3.6.3). The assembly processes of bacterial and fungal communities were evaluated by calculating the nearest taxon index and beta nearest taxon index (betaNTI) using the “picante” package. A betaNTI value < 2 indicates that the contribution is a stochastic process, and when betaNTI > 2 the shifts in community composition were considered to be shaped by deterministic processes.

The Phylogenetic Investigation of Communities by Reconstruction of Unobserved States (PICRUSt2) (Douglas et al., 2020) was applied to predict potential functional profiles of the bacterial community using 16S rRNA gene data.

## Data availability statement

The datasets presented in this study can be found in online repositories. The names of the repository/repositories and accession number(s) can be found below: https://www.ebi.ac.uk/ena, PRJEB56205.

## Author contributions

LL and HY conceived and designed the research. ZW, CF, JT, WW, DP, XD, HT and XZ conducted the experiment and analyzed the data. LL wrote the manuscript. All authors read and approved the manuscript.
